# Development of chronic actinic dermatitis in a patient with atopic dermatitis: a case report and a review of reported cases

**DOI:** 10.1093/skinhd/vzaf068

**Published:** 2025-10-09

**Authors:** Anri Kimura, Teruhiko Makino, Keita Takemoto, Shohei Kitayama, Megumi Mizawa, Tadamichi Shimizu

**Affiliations:** Department of Dermatology, Faculty of Medicine, Academic Assembly, University of Toyama, Toyama, Japan; Department of Dermatology, Faculty of Medicine, Academic Assembly, University of Toyama, Toyama, Japan; Department of Dermatology, Faculty of Medicine, Academic Assembly, University of Toyama, Toyama, Japan; Department of Dermatology, Faculty of Medicine, Academic Assembly, University of Toyama, Toyama, Japan; Department of Dermatology, Faculty of Medicine, Academic Assembly, University of Toyama, Toyama, Japan; Department of Dermatology, Faculty of Medicine, Academic Assembly, University of Toyama, Toyama, Japan

## Abstract

A 30-year-old Japanese woman who had suffered from atopic dermatitis (AD) since childhood was referred for the evaluation of itchy erythema in sun-exposed regions, which persisted for 3 years. Phototesting revealed that the minimal erythema dose (MED) to ultraviolet (UV)B radiation was 20 mJ cm^–2^ and MED to UVA radiation was 10 J cm^–2^. Phototesting with visible light revealed no positive reaction. The skin lesion was diagnosed as chronic actinic dermatitis (CAD). She was asked to strictly avoid exposure to sunlight; thereafter, her skin symptoms disappeared. There have been 18 reported cases of CAD developing in patients with AD, including our patient. Interestingly, CAD appeared before the age of 30 years in 15 of the 18 cases, suggesting that the age of onset of CAD in patients with AD may be younger than that in typical patients with CAD. Accordingly, we recommend performing phototesting on patients with AD with features of photoaggravated dermatitis to diagnose photosensitivity and provide appropriate management.

## Teaser

A few patients with photoexacerbations of atopic dermatitis (AD) have complications of a photosensitive disorder. We experienced development of chronic actinic dermatitis in an AD patient. In addition, we review reported AD patients with chronic actinic dermatitis.

Atopic dermatitis (AD) is a chronic inflammatory skin disease, characterized by elevated interleukin (IL)-4 and IL-13 signatures and extensive barrier dysfunction due to filaggrin downregulation. Sunlight exposure causes deterioration of skin symptoms in up to 10% of patients with AD. However, most patients with AD with a history of photoexacerbations showed no reduction in minimal erythema dose (MED) to ultraviolet A (UVA) or ultraviolet B (UVB) in phototesting.^1^ This may be due to the aggravation of the underlying dermatitis caused by heat-induced sweating and/or sunburn, whereas previous reports have demonstrated that some patients with photoexacerbations of AD have complications of a photosensitive disorder, such as polymorphic light eruption, actinic prurigo or drug-induced phototoxicity.^2^ Among patients with AD with a photosensitive disorder, the incidence of chronic actinic dermatitis (CAD) is estimated to be 3–10%.^1^ CAD is a photosensitive disorder that typically develops in older men, and shows a reduced MED to UVA, UVB and/or visible light. Herein, we report a case of CAD in a patient with AD and review the previous literature.

## Case report

**Figure 1:**
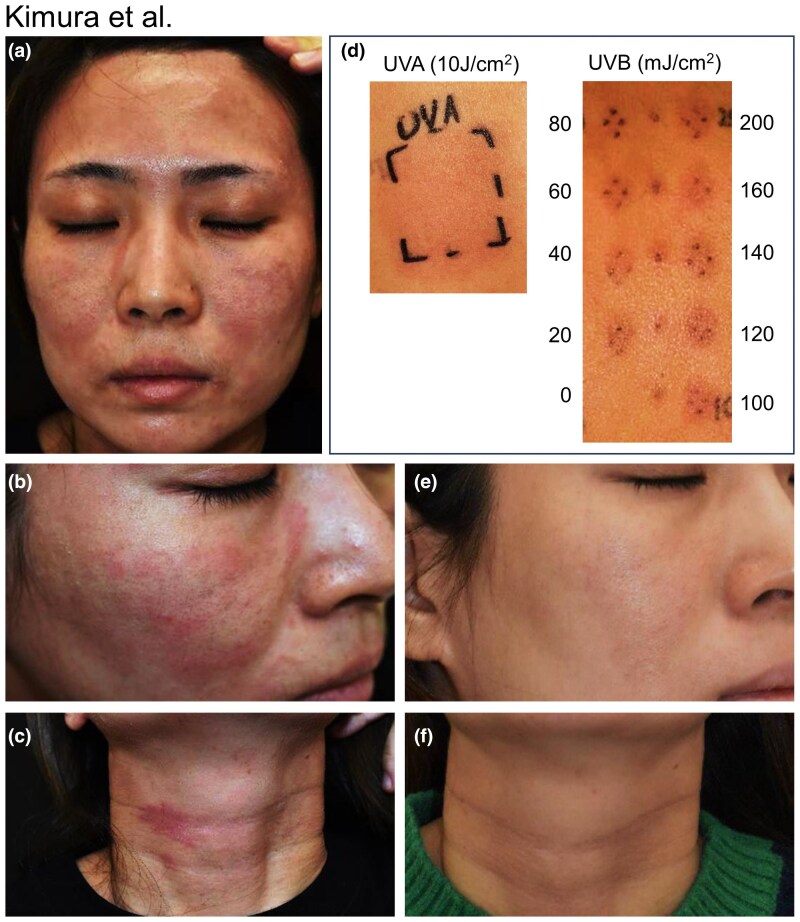
(a) Clinical appearance. Erythema on the face. (b, c) Eczematous plaques on the (b) right cheek and (c) neck. (d) Phototesting results. (e, f) Improvement of erythema and eczematous plaque on the (e) right cheek and (f) neck approximately 6 months after strict sun protection.

A 30-year-old Japanese woman was referred to our hospital for evaluation of skin lesions in sun-exposed regions. She had a history of AD since childhood. The patient had no relevant medical or family history. She was not taking any medication. Her Fitzpatrick skin phototype was III. Her skin symptoms were well controlled with topical steroid treatment throughout the year. She always used sunscreen when she went out and had never experienced skin flare-ups after sun exposure. However, approximately 3 years previously, the patient had noticed worsening skin lesions on her face, neck, forearms and dorsal sides of the hands after exposure to sunlight. This symptom was markedly enhanced during summer. At presentation, erythema and eczematous plaques with itching were observed on the forehead, eyelids (Figure 1a), cheeks (Figure 1b), neck (Figure 1c) and dorsal sides of the hands, despite usually using sunscreen. Phototesting revealed that the MED to UVB radiation using a PL-L36W UV6 lamp (wavelength 280–360 nm; Koninklijke Philips, Amsterdam, the Netherlands) was 20 mJ cm^–2^ (normal range 50–150) and the MED to UVA radiation using a PL-L36W/09 UVA lamp (wavelength 320–410 nm; Koninklijke Philips) was 10 J cm^–2^ (normal range >10) (Figure 1d). Phototesting of visible light using a slide projector with a 380-W bulb at a distance of 15 cm revealed no positive reaction at either 5 min or 24 h after 10 min of exposure. Blood test results showed increased levels of IgE (5248 UA mL^–1^; normal range <170) and thymus and activation-regulated chemokine, a marker for assessing the severity of AD (854 µg dL^–1^; normal range <450). The erythrocyte-free protoporphyrin concentration was 29 μg dL^–1^ (normal range 30–86). Tests for antinuclear, anti-dsDNA and anti-SS-A antibodies were negative. Other results were within normal limits. Patch test of the Japanese baseline series using PATCH TEST PANEL(S) (Sato Pharmaceutical Co., Tokyo, Japan) showed a positive reaction (+) to gold according to International Contact Dermatitis Research Group's criteria, although she had no experience of contact allergies to gold because she has not worn gold jewellery. Accordingly, the skin lesion that developed after sunlight exposure was diagnosed as CAD. The patient enacted strict protection from sunlight using a wide-brimmed hat, long-sleeved clothes and sunscreen, in addition to topical steroid treatment. Approximately 6 months later, her facial and neck skin symptoms disappeared (Figure 1e, f).

## Discussion

In this report, we demonstrated that CAD developed on the face, neck and dorsal sides of the hands of a patient with AD. CAD is characterized by an eczematous eruption on sun-exposed regions, usually in older men, and may be a contact dermatitis-like reaction to an endogenous cutaneous antigen, presumably induced by UV exposure.^3^ CAD typically persists for years, after which it frequently disappears. Lymphomatous malignant transformation has also been reported, possibly as a result of continued antigenic stimulation in long-standing active CAD, although it is extremely rare.^3^ We reviewed previously reported cases of CAD in patients with AD. To our knowledge, there have been 18 reported cases (in 10 male patients and 8 female patients) in 7 studies, including the present case (Table 1).^4–10^ Of the 18 patients with CAD, 7 were White and 11 were Asian. No information regarding the Fitzpatrick skin types of these patients was provided; therefore, the correlation between the development of CAD and skin phototype is unclear. In all patients, AD was recognized from infancy or childhood; thereafter, photosensitivity due to CAD appeared. Interestingly, CAD appeared at <30 years of age in 15 of the 18 patients, and at 31–40 years of age in the others. This finding indicates that the age of onset of CAD in patients with AD seems to be younger than in typical patients with CAD, although the reason for this is unclear. A marked reduction in MED to UVB was observed in all patients. A reduction in MED to UVA and a positive reaction to visible light were observed in 16 and 6 of the 18 patients, respectively. In addition, patients with CAD are known to have multiple contact allergies, as well as photosensitivity, chiefly to sesquiterpene lactone, colophony and fragrances.^11^ Fifteen patients with CAD and AD underwent patch testing using the European Standard Series, medicaments, *Compositae oleoresins* and other tests. Consequently, 13 of the 15 patients showed a positive reaction to the antigens. However, the detected antigens varied, including metals, fragrances and organic solvents, and there appeared to be no correlation between the antigens and CAD. Photoprotection is essential for the management of CAD. Sunlight avoidance, and use of sunscreens or skin-protective clothing were recommended. In three previous reports, a description regarding the treatment and outcome of CAD symptoms was not found; however, in other reports, patients improved with the use of sunscreens and the administration of medications, such as ciclosporin or pimecrolimus. Recent studies have demonstrated the usefulness of dupilumab, methotrexate or Janus kinase inhibitors in severe cases.^12^ Interestingly, one patient (patient 15) showed spontaneous resolution of photosensitivity 5 years after the onset of CAD, although the mechanism of this was unclear.^8^

**Table 1:** Characteristics of reported patients (*n* = 18) with chronic actinic dermatitis (CAD)

Patient	Age (years)	Sex	Race	Age at onset of AD (years)	Age at onset of photosensitivity (years)	MED at 24 h to UVA (J cm^–2^)	MED at 24 h to UVB (mJ cm^–2^)	Reaction to visible light	Patch tests	Treatments	CAD outcome	Reference
1	28	M	Asian	Childhood	28	10 (normal >10)	7.2 (normal 18–72)	Positive	Thimerosal *p*-phenylenediamine, formalin	Topical corticosteroid, sunscreen	Worsening	Kurumaji *et al*.^4^
2	28	F	White	Infancy	23	365 ± 30 nm: 2.7^a^ (normal >8.2)	305 ± 5 nm: <1.8^a^ (normal >27)	Positive	Amerchol 101	n.d.	n.d.	Russell *et al*.^5^
3	22	M	Asian	21	21	365 ± 30 nm: 2.2^a^ (normal >8.2)	305 ± 5 nm: <1.5^a^ (normal >27)	Negative	Chrome, cobalt, nickel, wool alcohol	n.d.	n.d.	
4	21	M	Asian	Infancy	18	365 ± 30 nm: 2.7^a^ (normal >8.2)	305 ± 5 nm: 3.9^a^ (normal >27)	Positive	Chrome	n.d.	n.d.	
5	21	M	Asian	Infancy	15	365 ± 30 nm: 1.5^a^ (normal range: >8.2)	305 ± 5 nm: 2.7^a^ (normal range: >27)	Negative	Fragrance mix, colophony, formaldehyde	n.d.	n.d.	
6	9	M	White	Infancy	9	365 ± 30 nm^a^: 4.7^a^ (normal range: >8.2)	305 ± 5 nm: 8.2^a^ (normal range: >27)	Negative	Negative	n.d.	n.d.	
7	23	M	Asian	Infancy	22	365 ± 30 nm: 1.0^a^ (normal range: >8.2)	305 ± 5 nm: 1.0^a^ (normal range: >27)	Negative	Fragrance mix, colophony, daisy	n.d.	n.d.	
8	27	M	Asian	9	17	365 ± 30 nm: 1.2^a^ (normal range: >8.2)	305 ± 5 nm: 3.9^a^ (normal range: >27)	Negative	Daisy, Tansy	n.d.	n.d.	
9	41	F	White	Childhood	38	Reduced	Reduced	n.d.	Perfume mix	n.d.	n.d.	Creamer *et al*.^6^
10	24	F	Asian	Childhood	20	n.d.	Reduced	Positive	Negative	n.d.	n.d.	
11	49	F	White	20	20	n.d.	Reduced	n.d.	Sesquiterpene lactone	n.d.	n.d.	
12	21	F	Asian	Childhood	21	Reduced	Reduced	Positive	Nickel, perfume mix	n.d.	n.d.	
13	17	F	Asian	Early childhood	7	350 ± 20 nm: 0.63^a^ (normal range: >10)	300 ± 5 nm: 2.5^a^ (normal range: >14)	n.d.	Negative	n.d.	n.d.	Ogboli *et al*.^7^
14	17	M	White	infancy	16	350 ± 20 nm: 20^a^ (normal range: >10)	300 ± 5 nm: 2.5^a^ (normal range: >14)	n.d.	Negative	n.d.	n.d.	
15	28	M	Asian	Infancy	25	335 ± 30 nm: 0.4^a^ (normal range: >1.8)	305 ± 5 nm: 3.9^a^ (normal range: >27)	n.d.	Potassium dichromate, aqueous cream, formaldehyde	Skin-protective clothing, use of museum film, sunscreens, ciclosporin, PUVA	Spontaneous resolution	Bryden *et al*.^8^
16	33	M	White	Childhood	32	360 ± 20 nm: negative^a^ (normal range: >8.2)	300 ± 5 nm: 10^a^ (normal range: >14)	Positive	Negative	Sunlight avoidance by shielding, mometasone furoate, pimecrolimus	n.d.	Yones *et al*.^9^
17	61	F	White	Childhood	36	20 (normal range: >10)	311 ± 2 nm: 75^b^ (normal range: >150)	Negative	Quaternium 15, balsam of Peru, colophony, propylene glycol, propolos	Sunscreens, ciclosporin	Partially improved	Quatrano *et al*.^10^
18	30	F	Asian	Childhood	27	10 (normal range: >10)	20 (normal range: 50–150)	Negative	Gold	Sunscreens	Improvement	Our patient

AD, atopic dermatitis; F, female; M, male; MED, minimal erythema dose; n.d., not described; PUVA, psoralen + UVA; UVA, ultraviolet A; UVB, ultraviolet B. ^a^MED to monochromator, ^b^MED to narrowband UVB.

In a retrospective review, Tan *et al*. demonstrated that 37.1% of 70 patients with CAD had a history of AD.^13^ In addition, this study showed that CAD presents with an earlier age at onset and an inverted male to female ratio in patients with darker skin types (phototypes V–VI) vs. lighter skin types (phototypes I–IV).^13^ Chaiyabutr *et al*. demonstrated that early-onset CAD (diagnosed at <40 years of age) was correlated with a higher rate of skin phototypes IV–VI (34.2% vs. 8.8%), AD (89.1% vs. 63.2%) and allergic rhinitis (87.1% vs. 58.3%) than late-onset CAD (diagnosed at ≥40 years of age) in a retrospective study of 130 cases of CAD.^14^ Furthermore, Chaiyabutr *et al*. established diagnostic categories; as photoexcerbated AD (normal phototesting), photosensitive AD (slight-to-moderate UVA sensitivity and slightly reduced UVB MEDs) and CAD (very low UVB MEDs and/or very low UVA MEDs) in patients with AD with suspected light exacerbations.^15^ These entities can change within patients over time.^15^

Occasionally, refractory facial redness or photoexacerbation of skin lesions is experienced. We hypothesized that CAD may be involved in these symptoms in patients with AD, even in young patients. Accordingly, we recommend performing phototesting on patients with AD with features of photo-aggravated dermatitis to diagnose photosensitivity and provide appropriate management.

## Data Availability

The data underlying this article will be shared on reasonable request to the corresponding author.
